# Imaging of human airways by endoscope-compatible dynamic microscopic optical coherence tomography

**DOI:** 10.3389/fmed.2025.1658890

**Published:** 2025-10-20

**Authors:** Cornelia Holzhausen, Hinnerk Schulz-Hildebrandt, Martin Ahrens, Noah Heldt, Mario Pieper, Heike Biller, Sönke von Weihe, David Ellebrecht, Mustafa Abdo, Stefan Steurer, Christoph Fraune, Klaus F. Rabe, Gereon Hüttmann, Peter König

**Affiliations:** ^1^Institute of Anatomy, University of Lübeck, Lübeck, Germany; ^2^Airway Research Center North (ARCN), German Center for Lung Research (DZL), Großhansdorf, Germany; ^3^Institute of Biomedical Optics, University of Lübeck, Lübeck, Germany; ^4^Wellman Center for Photomedicine, Massachusetts General Hospital, Harvard Medical School, Boston, MA, United States; ^5^LungenClinic Grosshansdorf, Großhansdorf, Germany; ^6^Department of Pathology, University Medical Center Hamburg-Eppendorf, Hamburg, Germany

**Keywords:** microscopic optical coherence tomography, virtual biopsy, airways, lung, tumor, cancer, inflammation

## Abstract

**Introduction:**

Microscopy is a cornerstone for diagnostics in lung disease but was traditionally restricted to biopsies and explanted tissue. Microscopic optical coherence tomography (mOCT) produces images with microscopic resolution without the need for exogenous markers. As recently demonstrated in excised mouse tissue, the combination with dynamic contrast (dmOCT) generates high contrast images of airway tissue. DmOCT therefore has the potential to be used for virtual biopsies in humans.

**Methods:**

To assess the potential of dmOCT combined with endoscopic imaging, we scanned excised human lung tissue through a custom-built endoscope optic and compared the resulting dmOCT images with conventional histologic sections of the same tissue. We also assessed if imaging time can be substantially reduced while keeping sufficient dmOCT image quality.

**Results:**

Endoscopic dmOCT successfully visualized the epithelium and subepithelial tissue of human airways including smooth muscle cells and glands. The technique detected key structural changes such as inflammatory cell infiltration, basement membrane thickening, epithelial damage, and the transition to carcinoma *in situ*. In addition, dmOCT distinguished between different morphologies of human lung cancer present in the examined tissue. The image contrast for discriminating these structures remained sufficient even after the acquisition time was reduced to 0.054s.

**Discussion:**

We have shown that dmOCT, when combined with endoscopic optics, reaches the image quality and imaging speed making its use for virtual biopsies *in vivo* realistic in the future.

## 1 Introduction

Microscopy is an indispensable tool for diagnostics and research in lung diseases. Traditionally, microscopy of human tissue was restricted to biopsies or explanted tissue. However, taking biopsies via a bronchoscope possesses the challenge to capture the region of interest (1). Therefore, a technology is needed to deliver images with high resolution and enable virtual biopsies. One promising technique is synchrotron-based imaging that has recently reached microscopic resolution in whole organs (2). If this technology will ultimately be usable in living humans remains to be determined and it is associated with radiation exposure (3). One step further toward virtual biopsies is endomicroscopy, a combination of bronchoscopy and confocal microscopy. Endomicroscopy has been used to visualize cells in the human lung *in vivo* (4, 5). By scanning the specimen, *en face* views of the tissue are generated (4). This technique relies on fluorescent dyes that are either present in the tissue or must be exogenously applied (4).

Optical coherence tomography (OCT) is also a point-scanning-based method that instead of fluorescence detects reflected light (6). In contrast to reflective confocal microscopy, it records reflection of the light from different tissue depths simultaneously. Hence, scanning a line with OCT results in a 2D cross sectional image. Image acquisition is therefore faster than confocal microscopy and results in imaging speeds that can exceed 100 frames/s (7). Using high numerical aperture (NA) optics, the resolution of OCT can be increased to microscopic resolution (mOCT) (8). Due to its speed, it can capture fast processes such as mucus transport in mice *in vivo* (9, 10). However, limited contrast, due to the speckle noise, hinders the ability to resolve single cells when imaging airways (11).

To overcome these limitations, additional contrast can be generated by analyzing dynamic fluctuations of mOCT signals over time that are induced by movement of intracellular organelles (12, 20). Our group has recently shown that dynamic mOCT (dmOCT) images allow the identification of different tissue components, including inflammatory cells in the airways of mice that could not be visualized by conventional mOCT (11). So far, this technology is only available on bench-top setups using high quality microscope objectives. Endoscopic mOCT without dynamic contrast has already been used to image the upper airways in humans (13, 14). The studies on upper airways focused on mucociliary transport. The endoscopes used in both studies have the resolution to reliably identify epithelial changes and the presence of inflammatory cells. However, the contrast achieved by conventional OCT is not sufficient to reliably identify these entities. The increased contrast of dmOCT offers the potential to identify tissue changes not visible by conventional mOCT.

Another challenge for high quality endoscopic images is motion of the tissue. Commonly, dmOCT images are calculated by analyzing 150 images acquired over 1.35 s. A time span in which motion of the tissue occurs and can interfere with dynamic contrast. For *in vivo* imaging, the image acquisition time must therefore be considerably reduced. Reducing the acquisition time substantially, however, could diminish the dynamic contrast.

To explore dmOCT's potential for endoscopic lung imaging, we investigated its ability to identify the morphology of the lung, the epithelium and the subepithelial tissue on surgically excised human lung specimens. We used an endoscope optic comparable to the one we had previously used for mOCT imaging of the nasal mucosa in humans (13). Criteria for the usability were, besides imaging of the morphology itself, the visibility of immune cells, of the basement membrane and of alterations in the tissue organization as the ones that occur in chronic inflammation or neoplasia. To counter the difficulties that are to be expected with tissue motion, we have reduced the imaging time and also evaluated the resulting image quality.

## 2 Materials and methods

### 2.1 Setup

**Figure 1:**
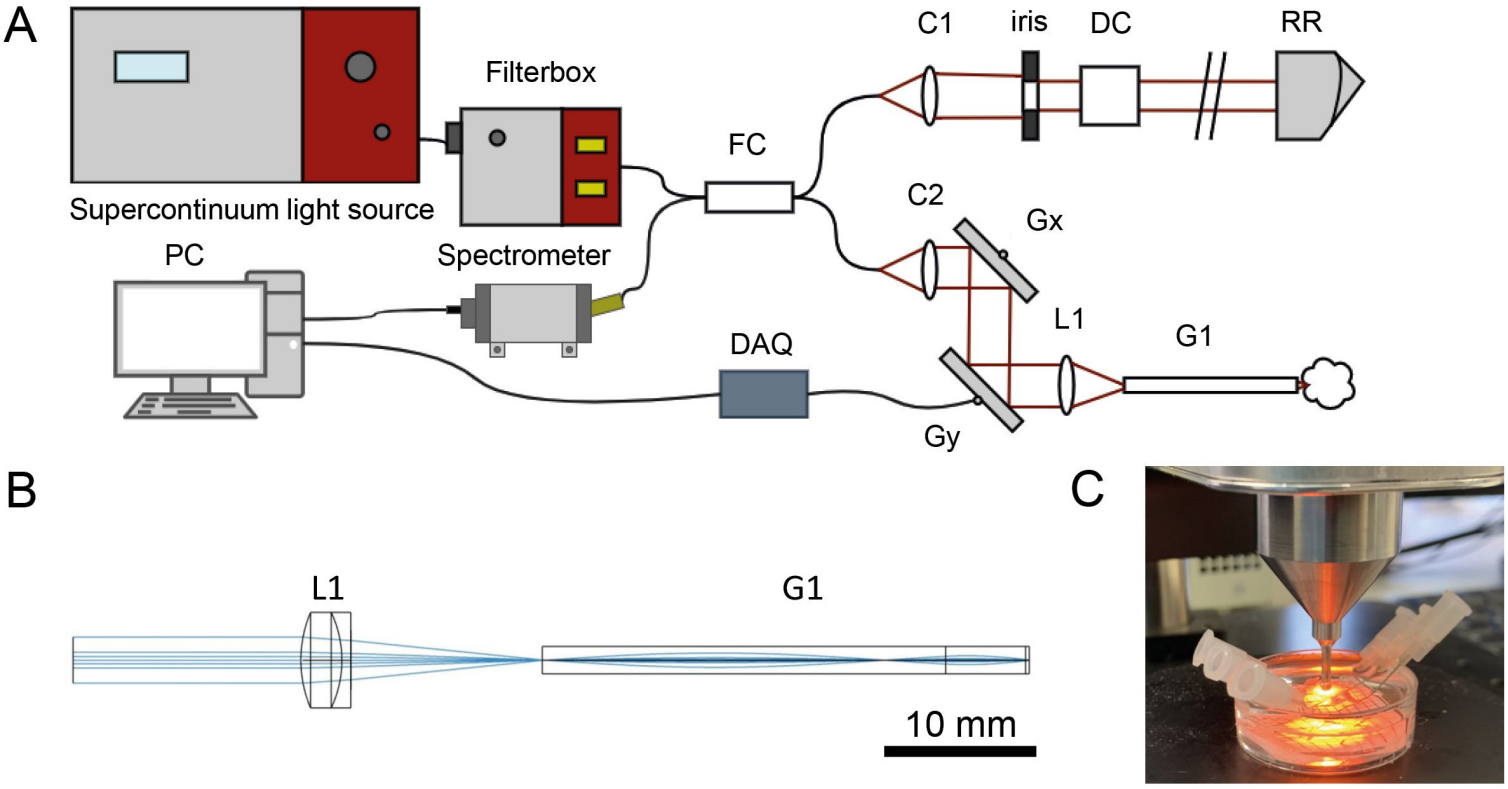
mOCT setup with imaging optics. **(A)** Schematics of the OCT device: FC: 50/50 fiber coupler; C1/C2, collimators; Gx, Gy, galvanometer mirror scanner; L1, focussing lenses; G1, GRIN lens system; DC, dispersion compensation; RR, retro-reflector; DAQ, data acquisition device; PC, computer for data acquisition and scanning control. **(B)** Beam path through the scan and GRIN optics, which has an outer diameter of 2.3 mm. **(C)** Preparation and immobilization of the tissue sample.

The principal setup (Figure 1A) is similar to the setup reported previously (20). In short, the system uses a broadband light source (SuperK Extreme EXW-4 OCT, NKT PHOTONICS A/S, Birkerød, Denmark) to achieve a high axial resolution. A filter box (SuperSplit, NKT PHOTONICS A/S) separates IR from the VIS/NIR light. The VIS light is coupled into an arm of a broadband fiber coupler (TW630R5A2, Thorlabs GmbH, Bergkirchen, Germany), which splits the light and guides it to a reference and a sample arm. The reference arm consists of a collimator, an iris diaphragm, dispersion correction and a retro-reflector. In the sample arm a collimator, a pair of galvanometric mirrors, and a self-designed scan optic, which includes the rigid endoscope, scan the tissue. The scan lens (49-305, Edmund Optics, U.S.) focuses the radiation into a combination of a low NA relay GRIN lens and a collimating GRIN rod lens (Figure 1B). The two GRIN lenses (relay lens: LFRL-180-025-20, length 26.556 mm, focus lens: LFRL-180-025-50, length 5.252 mm) were custom made (Grintech GmbH, Jena, Germany) and glued into a rigid steel tube with an outer diameter of 2.3 mm. To reduce parasitic reflections, the surfaces of the GRIN lenses were polished to 8°. The backscattered light from the sample and the reflected light of the reference path result in an interference signal, which is recorded by a custom-built high-speed spectrometer (Thorlabs GmbH, Bergkirchen, Germany) with a CMOS camera working at an A-scan rate of 100 kHz (OctoPlus CL, Teledyne, e2v, Canada).

In contrast to confocal or non-linear microscopy, in OCT the lateral and axial resolution are not directly coupled. The axial resolution Δ*z* is determined by the spectral width Δλ and the central wavelength λ of the spectrum used for determining the depth of the scattering structures [Δ*z* = (2ln 2/*n π*) λ^2^/Δλ; Fujimoto et al. (6)] and is, under the conditions used here, independent on the depth. The index of refraction *n* is in biological tissues about 1.35. In contrast, the lateral resolution Δ*x* depends, as in confocal microscopy, on the numerical aperture (NA) of the imaging system and reaches in the focal plane $\Delta x=\left(\sqrt{\ln\;2\;}/\pi \right)\frac{\lambda }{NA}$ (15). Outside the confocal range, which has a length of $b=\left(\frac{\pi }{2}\right)\;\frac{\Delta x^{2}}{\lambda }$, the resolution and the signal intensity decrease. However, layered structure can still be imaged with good quality due to the high dynamic range of OCT and the constant depth resolution.

The OCT device used the spectral range covering 550–950 nm. With an effective full width at half maximum of 160 nm in air an axial resolution of Δ*z* = 1.2 μm was measured and the axial field of view (FOV) was 592 μm, when imaging tissue with a refractive index of 1.35. A lateral resolution of only Δ*x* = 2.1 μm was determined at the focal plane using the 1951 USAF resolution test chart. This corresponds to an effective NA of 0.1μm. The usable lateral field of view (FOV) was 700 μm.

The interference spectra were recorded with 2048 pixel at 100 kHz readout frequency. B-scans consisting of 512 pixels in lateral direction and 1024 pixels axially were recorded the 111 Hz. A time sequence of 150 B-scans was measured in 1.35 s for evaluation of the signal fluctuations.

Hence, the frequency of signal fluctuations could be measured between 0 and 55.56 Hz with a resolution of 0.74 Hz (11). For each pixel, the absolute values of the OCT time series were Fourier transformed along the time axis and the integral amplitude of three frequency bands were calculated. The frequency bands were color-coded, whereby low frequencies (< 1.85 Hz) are represented by blue, medium frequencies (>1.85–7.78 Hz) by green, and high frequencies (>7.78–24.81 Hz) by the red channel composed into a single RGB image. To avoid registration artifacts and coloring artifacts by the surface of the grin lens, visible within the image data, only data below this surface was processed.

The choice of these three frequency bands is based on the successful imaging of mouse trachea as previously described (11) and was manually adapted for this setup. The image contrast was then enhanced using histogram matching for each channel. The target histogram to match onto was chosen to be the logarithmic standard deviation over the time axis of the time series. Afterwards contrast and brightness were manually adjusted to further enhance image quality. Finally, for display purposes, all data shown was cropped to square FOVs of 500 μm width and height.

It was further assessed if the number of images necessary to calculate dynamic contrast can be reduced while maintaining sufficient image quality to distinguish morphologic structures.

### 2.2 Specimens, OCT imaging and further processing

Freshly excised pulmonary tissue samples were obtained from 20 different patients who had lobecomy or pneumonectomy at the LungenClinic Grosshansdorf. The specimens of the peripheral lung were imaged within less than 2 h of surgical resection. The airways were freed from surrounding tissue, longitudinally opened and positioned with the luminal side upwards. Tumor tissue was removed from the lung sample and trimmed to fit in the petri dish. Each sample was placed in a silicone lined petri dish with the surface of interest facing upwards, immobilized with needles, and covered with HEPES-buffered Ringer solution. The Petri dish was provided with a grid for orientation. For imaging, the endoscope tip was positioned directly above the tissue (Figure 1C). The tissue was scanned by mOCT to find a region of interest. Then dynamic images of the selected region were acquired as described above. Throughout the measurements, the specimens were kept at room temperature.

After mOCT imaging, the samples were fixed in 4% buffered formalin while still positioned in the dish for further histological processing. The grid within the petri dish was used for orientation during trimming. After embedding the formalin fixed tissue samples in paraffin (formalin fixed paraffin embedded, FFPE), histological sections were cut at 5 μm and stained with hematoxilin and eosin (H&E). The mOCT and dmOCT images were compared to the H&E-stained tissue sections from the same tissue specimen. It is important to note that due to the inability to label the exact area within the specimen where the mOCT image was taken, the shown H&E sections will not fully correspond to the mOCT or dmOCT images shown.

The study was approved by the Ethics Committee of the University of Lübeck (reference number: 21-306). All examinations were carried out according to the principles of the Declaration of Helsinki.

## 3 Results

### 3.1 Imaging physiological airway architecture with dmOCT

**Figure 2:**
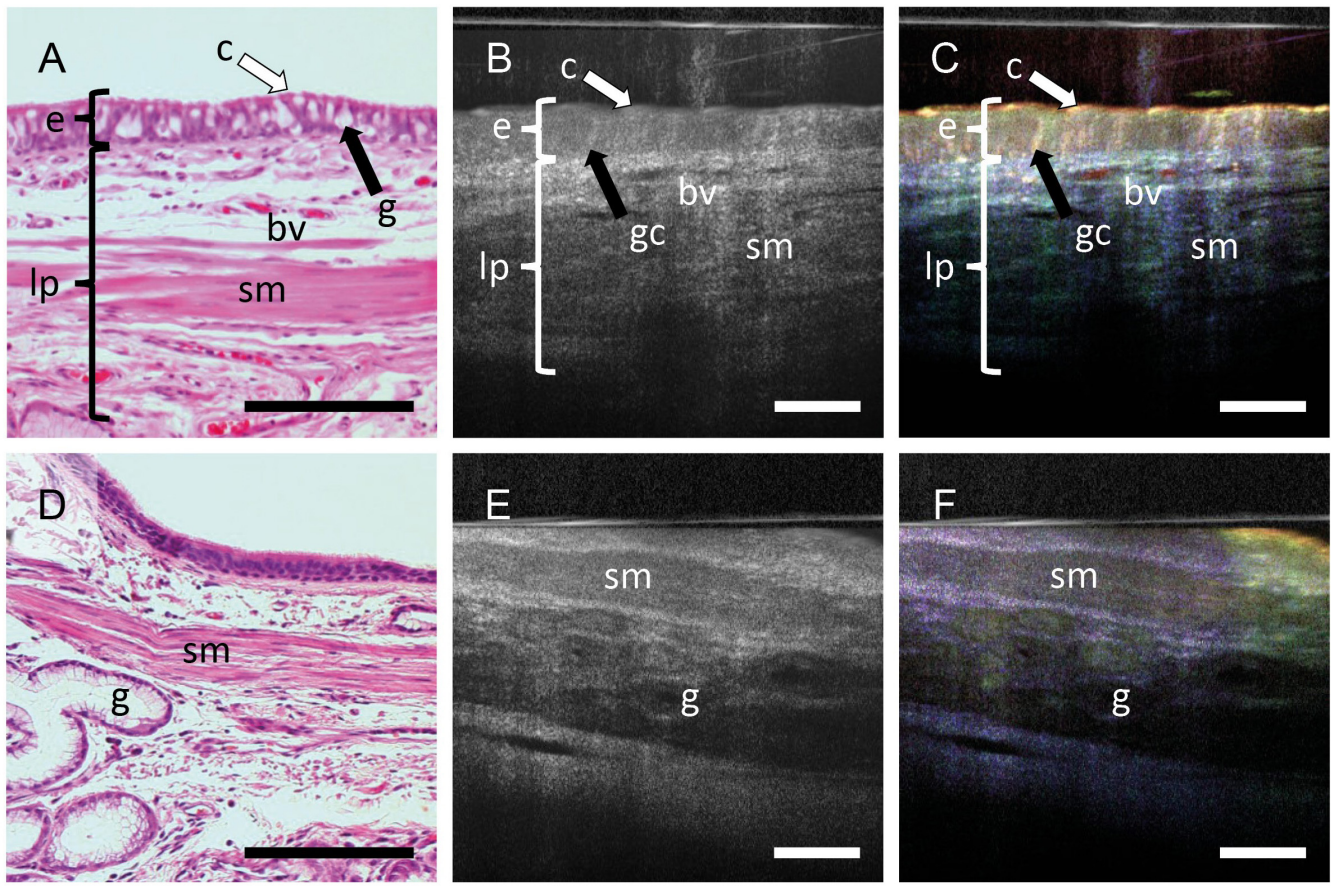
Dynamic contrast increases the information in mOCT images. FFPE sections stained with H&E **(A, D)** averaged mOCT **(B, E)**, and respective dmOCT images **(C, F)** form the corresponding. **(A–C)** mOCT imaging with focal plane within the epithelium and corresponding H&E-stained section. **(C)** Dynamic contrast allows to identify the epithelium (e) with cilia (c) and goblet cells (gc). In the lamina propria (lp), blood vessels (bv), smooth muscle (sm) can be detected. **(D–F)** When focusing on deeper layers by slightly compressing the tissue, smooth muscle (sm) and glands (g) can be identified within the connective tissue. Scale bar = 100 μm.

The histologic section obtained after mOCT imaging show the well-known morphology of human airyways (Figure 2A). The epithelium with ciliated and non-ciliated cells can be discriminated. In the subepithelial connective tissue, extracellular fibers, small blood vessels and smooth muscle cells are readily identified. In conventional averaged mOCT scans (Figure 2B) of the same specimen, the epithelium can be recognized by having less signal compared to the underlying connective tissue. Nevertheless, discriminating cell types is not possible and identifying subepithelial structures such as the extracellular fibers, blood vessels and smooth muscle cells is difficult. However, if dynamic contrast is used, the epithelium is easily recognizable by its greenish yellow color, representing motion of intracellular organelles, and ciliated and non-ciliated cells can be discriminated by the strong orange color originating from the fast coordinated motion of the cilia (Figure 2C). The subepithelial extracellular fibers, which do not actively move are visualized in blue color and can be distinguished from the epithelium. The blood vessels can be recognized by a strong red signal derived from the Brownian motion of intraluminal blood components. An increased OCT signal can be measured from deeper tissue structures, when the tip of the endoscopic probe slightly compresses the tissue and deeper focusing is possible (Figures 2D–F). While subepithelial glands and smooth muscle cells do not have a strong dynamic mOCT signal, the color contrast still distinguishes them from the surrounding connective tissue. During histological processing, the tissue experiences considerable shrinkage. To depict the same tissue area in OCT scans and histologic images, the histologic images were adjusted in magnification accordingly. This results in scale bars of different length in histologic and mOCT images.

### 3.2 Imaging pathologic alterations in airway morphology by dmOCT

**Figure 3:**
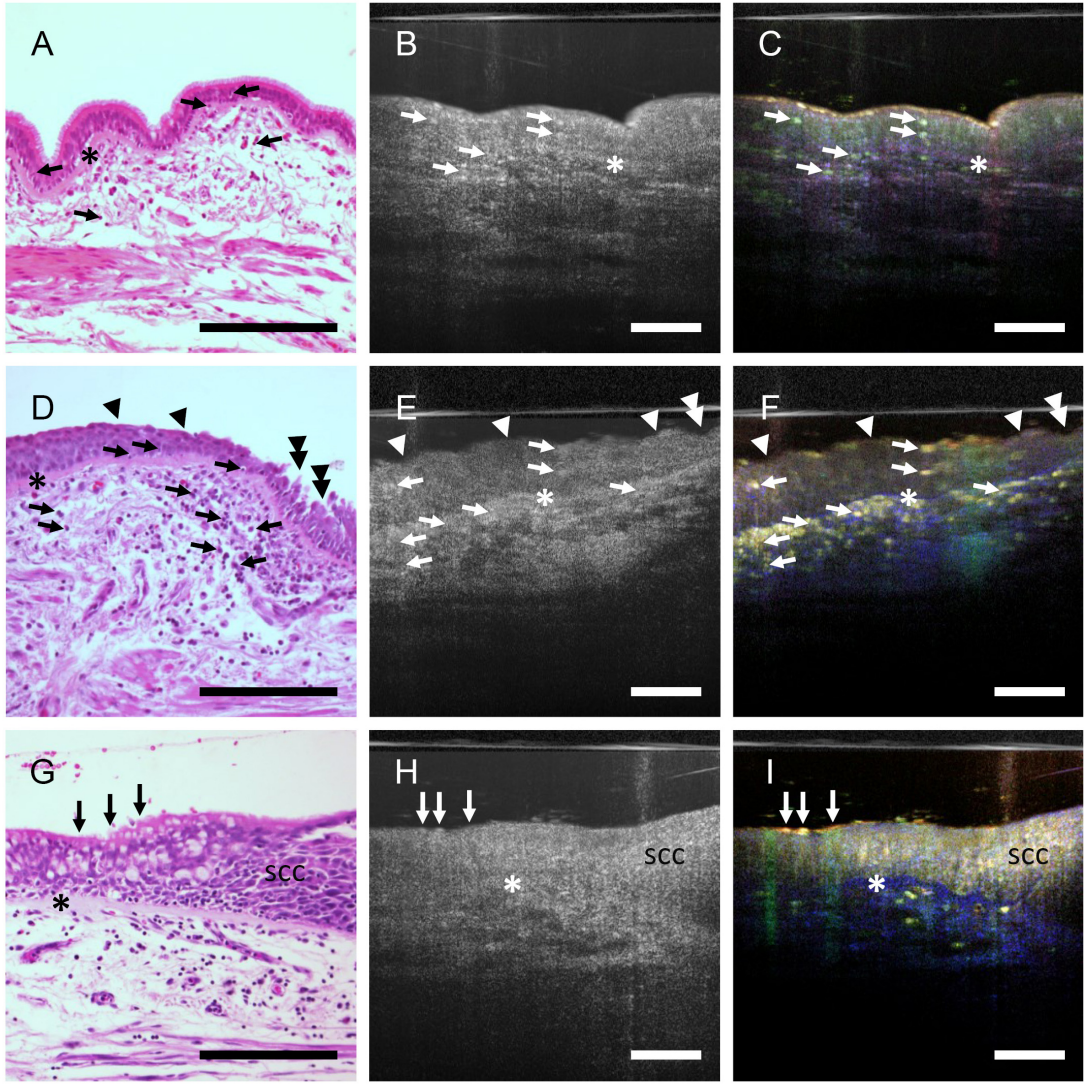
Inflammation and epithelial alterations in airways. FFPE-section after staining with H&E **(A, D, G)**, averaged mOCT images **(B, E, H)**, and respective dmOCT images **(C, F, I)** from the corresponding specimen. **(A–C)** Low grade inflammation with few intraepithelial and subepithelial immune cells (arrows) and thickened basement membrane (asterisk). The epithelial structure is preserved. **(D–F)** High-grade inflammation with numerous infiltrated immune cells (arrows), prominent thickening of the basement membrane (asterisk), multifocal loss of cilia (arrowheads) and epithelial damage (doubled arrowheads). **(G, H)** Loss of the regular architecture of the epithelium progressing from left to right, marking the transition from ciliated epithelium (arrows) to a squamous cell carcinoma *in situ* (SCC). Thickened basement membrane (*). Scale bar = 100 μm.

We have previously shown in the airways of mice that dmOCT with a bench top set up can detect immune cells with excellent contrast that were not visible with mOCT alone (11). With the endoscopic optic (Figures 3B–I), we could now also detect isolated cells with intense dynamic signal in human airway epithelium (Figure 3C) and connective tissue (Figure 3F). The presence of inflammatory cells in these areas was confirmed by histologic sections derived from the same specimen (Figures 3A, D). In some specimens the airway epithelium was segmentally reduced in thickness, had a weaker dynamic signal and loss of cilia activity (Figure 3F). Indeed, damage of the epithelium was confirmed in histologic sections (Figure 3D). In another specimen we also detected an increased thickness of the airway epithelium and an alteration of the normally ordered epithelial architecture which is histologically consistent with neoplastic growth (Figures 3G–I). A homogenous structure between the epithelium and the subepithelial connective tissue, which is present in dmOCT images from specimens with chronic inflammation (Figures 3C, F, I), was found to be a thickened basement membrane in histology (Figures 3A, D, G).

**Figure 4:**
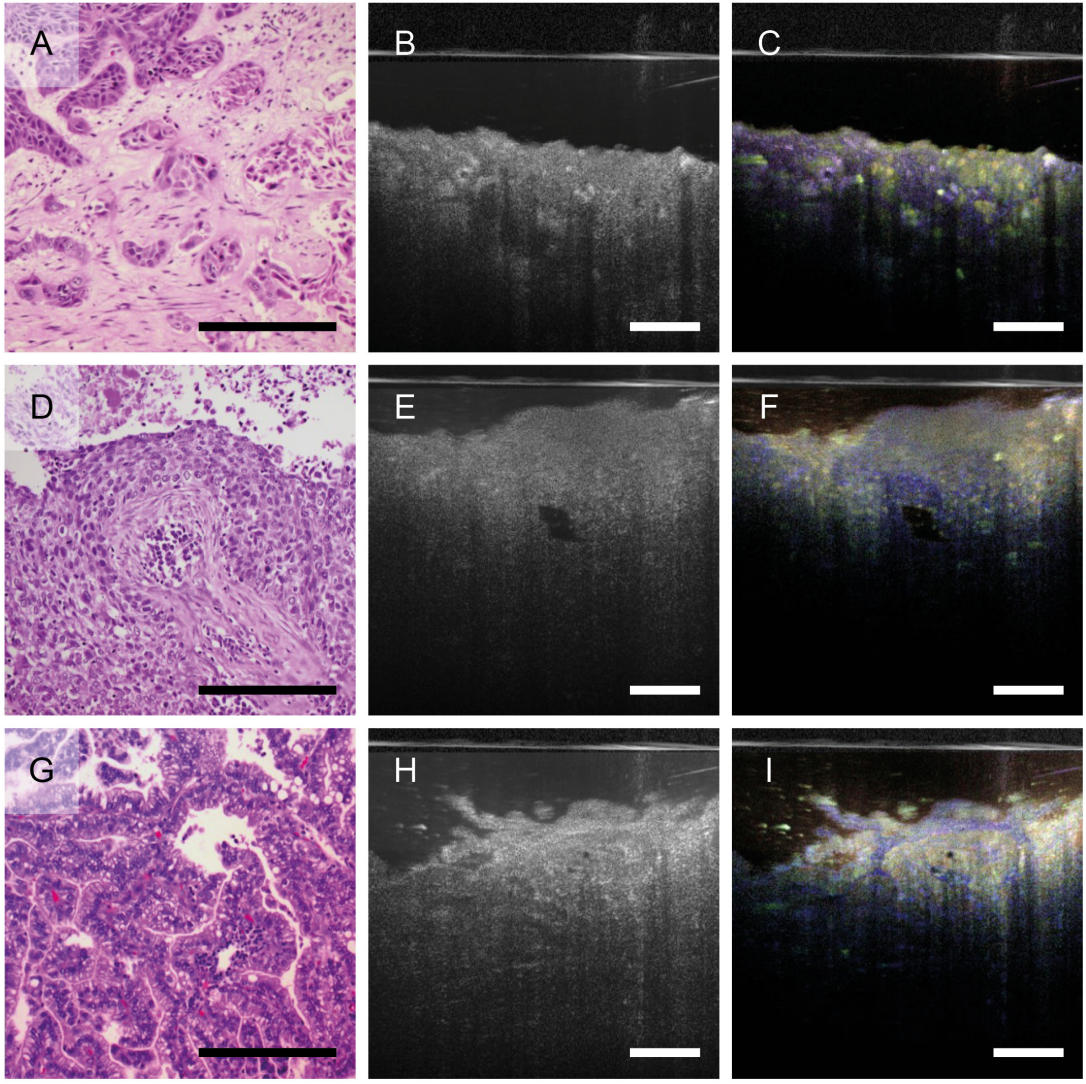
Growth pattern of different lung tumors imaged by dmOCT. H&E-stained FFPE-sections **(A, D, G)**, averaged mOCT **(B, E, H)**, and respective dmOCT images **(C, F, I)** from the corresponding specimen. **(A–C)** Squamous cell carcinoma with nests of neoplastic cells and abundant amount of desmoplastic stroma. **(D–F)** Squamous cell carcinoma with basaloid appearance. **(G–I)** Metastasis of a colon carcinoma growing in a glandular pattern. Scale bar = 100 μm.

Since dmOCT was able to detect changes in epithelial morphology and lung cancer is an important and frequent disease of the airways, we further investigated whether dmOCT can in principle differentiate between different morphological characteristics of cancers found in the lung. We compared histologic sections and dmOCT-images of squamous cell carcinomas, two of them shown here, and a lung metastasis of a colon carcinoma. These tumors exhibited different neoplastic cells to stroma ratio and growth patterns. The images obtained with dmOCT correlated well with the morphology of the respective tumor as observed in conventional H&E-stained sections (Figure 4).

### 3.3 Reduction of acquisition time of dmOCT

**Figure 5:**
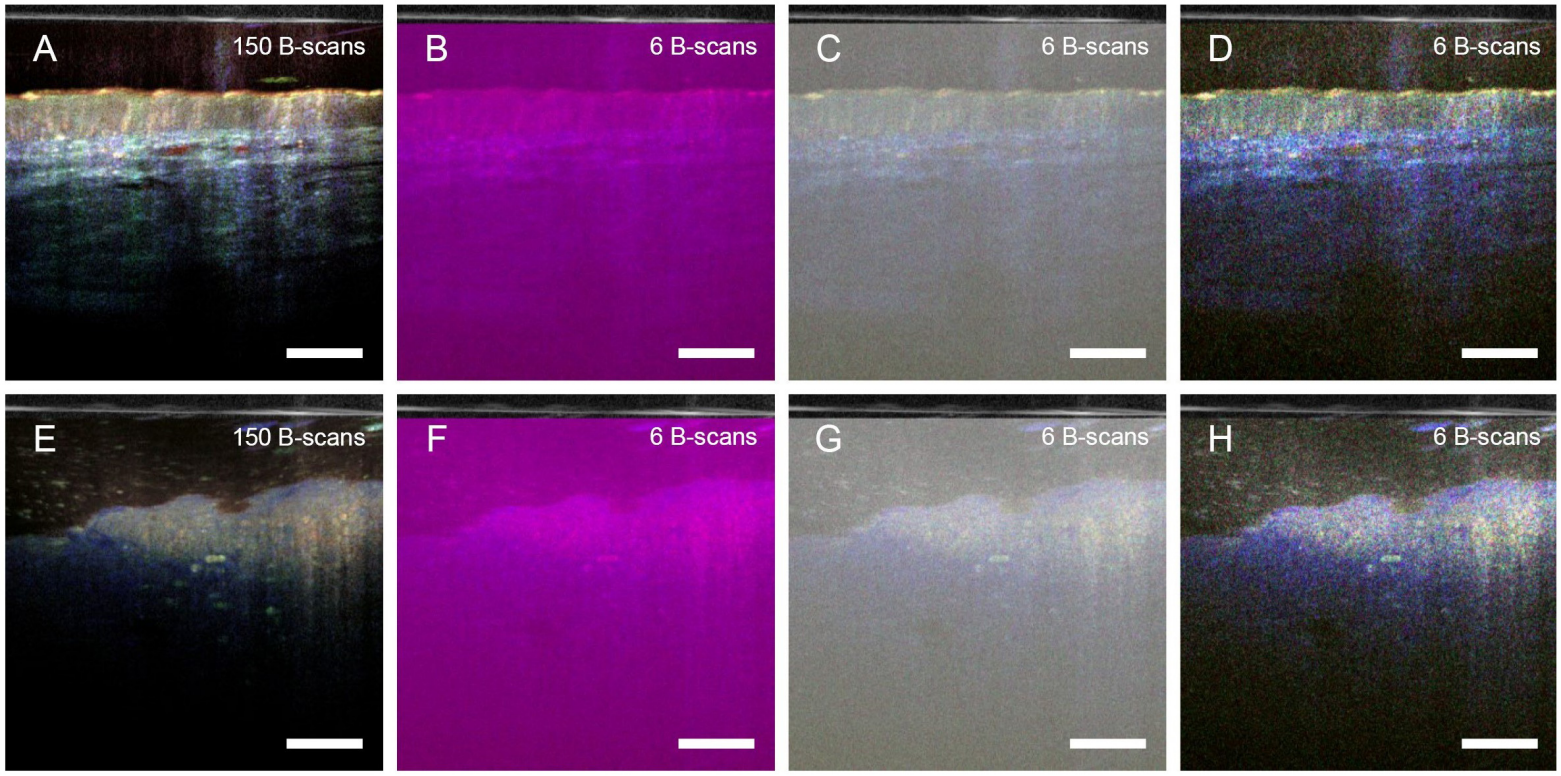
Reduction of acquisition time for dmOCT. **(A–D)** Human airway, **(E–H)** lung tumor. Images in **A** and **E** were calculated from 150 images, imaged in 1.35 s. **(B–D)** and **(G, H)** were calculated from six images imaged in 0.054 s. A-B and E-F were calculated with frequency bands 0–1.5 Hz for the blue channel, >1.5–7.5 Hz for the green channel and >7.5–25 Hz for the red channel resulting in loss of color information in **(B)** and **(F)**. **(C, D**) and **(G, H)** were calculated with manually adjusted frequency bands 0–9.3 Hz (blue), >9.3–27.8 Hz (green) and >27.8 Hz-46.3 Hz (red). For **D** and **H** additional contrast adjustment was performed manually. Scale bar = 100 μm.

One current drawback of dmOCT is its long acquisition time. The dmOCT images shown in Figures 2–4 were calculated from 150 images recorded in 1.35 s. This imaging time is too long for *in vivo* endoscopic imaging. Obviously, image quality and information depend on the number of images. To assess if sufficient image quality could be achieved within a shorter acquisition time, we calculated the dmOCT images with a lower number of frames. Compared to the original image calculated from 150 images (Figure 5A), a reduction of the sequence length to 6 frames (acquisition time: 0.054 s, 18.52 frames/s) reduces the spectral resolution to 18.5 Hz. Therefore, without adapting the frequency borders, the color channels are no longer correctly defined, and the resulting images are mainly purple colored (Figure 5B). Manual adaptation of the frequency bands to 0–9.3 Hz (blue), >9.3–27.8 Hz (green), and >27.8 Hz-46.3 Hz (red) restored visually similar color information by ensuring that each frequency range contained a bin of the Fourier transform. However, the reduced number of frames leads to an increased background brightness, lower contrast and more noise (Figure 5C). The image quality was further improved by manually adjusting contrast and brightness (Figure 5D), although the noise level was still higher, compared to the original image. Nevertheless, most structures that were visible in the original image could still be discriminated after reducing the acquisition time by a factor of 25. The same modified frequency bands could subsequently be used to analyze dmOCT images of tumor tissue (Figures 5E–H).

## 4 Discussion

We demonstrated that dmOCT with endoscopic optics provides cross-sectional images of explanted human lung tissue in which morphological features can be detected that are also identified in conventional histologic sections of the same tissue. Specifically, we were able to visualize epithelial morphology, the basement membrane, smooth muscle cells, blood vessels, glands, and inflammatory cells within different compartments of the tissue. These parameters can be used to detect tissue damage and inflammation of the airways. In addition, we were able to identify epithelial changes made by a carcinoma *in situ* and characterize the different growth patterns of tumors in the lung. We used optics very similar to those we have previously found to be useful for *in vivo* imaging of human nasal airways by mOCT (13) and showed that these optics also work for dynamic contrast. In principle the adaptation of dmOCT to endoscopic imaging of human airways seems therefore feasible and promising.

Compared to synchrotron-based imaging or endomicroscopy, at the present state of knowledge, no side effects are to be expected. Since the mOCT is based on the reflection of light, it does neither require fluorophores, which are necessary for endoscopic confocal imaging nor radiation exposure. The risk of tissue damage by potentially harmful reactive oxygen species or radiation is therefore greatly reduced (16, 17). Moreover, compared to confocal reflection microscopy, high speed image generation of dmOCT combined with the output of cross-sectional images makes dmOCT an ideal method for *in vivo* imaging. One major disadvantage of dmOCT, however, is the need to record multiple images to calculate the dynamic contrast which makes it prone to motion artifacts. Up to now we have used 150 frames, recorded in 1.35 s, to generate the images shown in Figures 2–4. This is obviously too long for intravital imaging of an organ that, like the lung, is constantly in motion. By reducing the number of frames for the dmOCT contrast calculation to six frames, the recording time is reduced to 0.054 s This results in 18.5 frames/s for dmOCT, which is 75% of the traditional video rate of 24 Hz and seven times faster than the recently published work where 6 frames over a time course of 375 ms where used (13).

In the approach we used, reducing the imaging time for dmOCT contrast calculation required manual determination of the cut offs of the frequency bands and image optimization to prevent color shifts. Manual determination, as done in this study, is not feasible for endoscopic imaging in the clinical setting. To solve this problem, we are currently working on an automated determination of the optimal frequency bands for dmOCT contrast.

An additional, more far-reaching method to overcome artifacts by movement, is through mathematical registration. Up to now, only movements within the acquisition plane can be corrected. With registration, movements that are orthogonal to the acquisition plane could be compensated as well. For this, faster mOCT systems that scan 3D volumes (7) are needed. With 3D volumes, movements in all spatial directions can be corrected. The capacity to compensate substantial movement artifacts by registration has already been demonstrated with full field OCT systems. These systems were able to resolve single photoreceptors in the human eye that was not immobilized (18). Currently, this approach is not available for endoscopic imaging. Another challenge that must be overcome for *in vivo* lung imaging is the compatibility of the method with flexible endoscopes. Recently, flexible endoscope designs were published which incorporated high numerical optics that should be compatible with full field OCT imaging (19). It is therefore conceivable that flexible endoscopes with microscopic resolution and dmOCT contrast will be available in the future.

In summary, many technological developments have substantially improved OCT in recent years. With faster imaging and new flexible endoscopes with high numerical optics on the horizon, endoscopic dmOCT has with these future adjustments the potential to be used as a bronchoscopy-based tool. This may improve our understanding of pathophysiological processes in the lung such as inflammation and lung cancer and possibly lead to new treatment options.

## Data Availability

The raw data supporting the conclusions of this article will be made available by the authors, without undue reservation.
